# Phenotypic Description of A Patient with ODLURO Syndrome and Functional Characterization of the Pathogenetic Role of A Synonymous Variant c.186G>A in *KMT2E* Gene

**DOI:** 10.3390/genes15040430

**Published:** 2024-03-29

**Authors:** Mario Benvenuto, Sofia Cesarini, Giulia Severi, Enrico Ambrosini, Angelo Russo, Marco Seri, Pietro Palumbo, Orazio Palumbo, Marco Castori, Emanuele Panza, Massimo Carella

**Affiliations:** 1 Division of Medical Genetics, Fondazione IRCCS-Casa Sollievo della Sofferenza, 71013 San Giovanni Rotondo, Italy; m.benvenuto@operapadrepio.it (M.B.); p.palumbo@operapadrepio.it (P.P.); o.palumbo@operapadrepio.it (O.P.); m.castori@operapadrepio.it (M.C.); 2 Dipartimento degli Studi Umanistici, Università degli Studi di Foggia, 71122 Foggia, Italy; 3 U.O.C. Genetica Medica, IRCCS Azienda Ospedaliero-Universitaria di Bologna, 40138 Bologna, Italy; sofia.cesarini@studio.unibo.it (S.C.); giulia_severi@aosp.bo.it (G.S.); enrico.ambrosini@unipr.it (E.A.); 4 U.O.C. Neuropsichiatria Infantile, IRCCS Istituto delle Scienze Neurologiche di Bologna, 40139 Bologna, Italy; russo.neuroped@gmail.com; 5 Medical Genetics Unit, IRCCS Azienda Ospedaliero-Universitaria di Bologna, 40138 Bologna, Italy; marco.seri@unibo.it (M.S.); emanuele.panza@unibo.it (E.P.); 6 Dipartimento di Scienze Mediche e Chirurgiche, Università di Bologna, 40126 Bologna, Italy

**Keywords:** *KMT2E*, splicing, neurodevelopmental disorders

## Abstract

O’Donnell-Luria-Rodan (ODLURO) syndrome is an autosomal dominant disorder caused by mutations in the *KMT2E* gene. The clinical phonotype of the affected individuals is typically characterized by global developmental delay, autism, epilepsy, hypotonia, macrocephaly, and very mild dysmorphic facial features. In this report, we describe the case of a 6-year-old boy with ODLURO syndrome who is a carrier of the synonymous mutation c.186G>A (p.Ala62=) in the *KMT2E* gene, predicted to alter splicing by in silico tools. Given the lack of functional studies on the c.186G>A variant, in order to assess its potential functional effect, we sequenced the patient’s cDNA demonstrating its impact on the mechanism of splicing. To the best of our knowledge, our patient is the second to date reported carrying this synonymous mutation, but he is the first whose functional investigation has confirmed the deleterious consequence of the variant, resulting in exon 4 skipping. Additionally, we suggest a potential etiological mechanism that could be responsible for the aberrant splicing mechanism in *KMT2E*.

## 1. Introduction

O’Donnell-Luria-Rodan (ODLURO) syndrome (OMIM:618512) is an autosomal dominant disorder, described for the first time in 2019 by O’Donnell-Luria and collaborators [1].

It is caused by mutations in the *KMT2E* gene (*MLL5*) located on chromosome 7, which encodes a lysine N-methyltransferase able to recognize and selectively bind to H3K4me3 and H3K4me2 methylated histones, where it may regulate transcription factor binding or otherwise regulate transcription. *KMT2E* has been reported to play key roles in diverse biological processes, including cell cycle progression, genomic stability maintenance, adult hematopoiesis, and spermatogenesis. In addition, it has been suggested to interact with NCOR2 and TBL1X in a repressor complex regulating cytokinesis-associated genes [2,3].

*KMT2E* is part of the KMT2 family, but it is set apart from other gene family members because it contains only one N-terminal SET domain and does not possess intrinsic catalytic activity, lacking intrinsic histone methyltransferase (HMT) activity towards histone substrates [2].

Other members of this family have also been associated with neurodevelopmental disorders; those conditions include Wiedemann-Steiner syndrome (OMIM:605130; *KMT2A*/*MLL1*), childhood-onset dystonia (DYT28) (OMIM:617284; *KMT2B*/*MLL2*), Kleefstra syndrome-2 (OMIM:617768; *KMT2C*/*MLL3*), Kabuki syndrome type 1 (OMIM:147920; *KMT2D*/*MLL4*), epilepsy, early-onset, type 2, with or without developmental delay (OMIM:618832; *KMT2D*/*MLL4*), neurodevelopmental disorder with speech impairment and dysmorphic facies (OMIM:619056; *KMT2F*/*SETD1A*), and, finally, intellectual developmental disorder with seizures and language delay (OMIM: 619000; *KMT2G*/*SETD1B*).

The main clinical characteristics of ODLURO syndrome are global developmental delay, autism, epilepsy, hypotonia, gastrointestinal issues, macrocephaly, and mild dysmorphisms [4]. The majority of the causative variants are de novo and are more frequently detected in males.

In this report, we describe the case of a male proband that presents ODLURO syndrome caused by the c.186G>A (p.Ala62=) synonymous variant in the *KMT2E* gene, and the results of the functional study to prove its effect on altering the splicing. The variant had already been reported in a patient with similar characteristics [5], but the pathogenic effect was not confirmed by functional analysis so far.

## 2. Materials and Methods

### 2.1. Genomic DNA Extraction and Quantification

Peripheral blood samples were obtained from both the proband and his parents while genomic DNA was isolated by using Bio Robot EZ1 (Qiagen, Solna, Sweden). The quality of nucleic acid was tested on a 1% electrophoresis agarose gel, and the concentration was obtained by using a Nanodrop 2000 C spectrophotometer (Thermo Fisher Scientific, Waltham, MA, USA). The family provided written informed consent to molecular testing and to the full content of this publication and the study was approved by the Casa Sollievo della Sofferenza Hospital ethics committee (protocol No. 177CE). 

### 2.2. Next-Generation Sequencing Analysis

Targeted resequencing (TRS) on proband DNA was performed using a SureSelect gene panel (Agilent Technologies, Boulder, CO, USA) designed to selectively capture 338 known genes associated with syndromic and non-syndromic forms of neurodevelopmental disorders. Libraries were prepared using the SureSelect target enrichment kit (Agilent Technologies, Boulder, CO, USA) following the manufacturer’s instructions. Subsequently, the targeted fragments obtained were sequenced on a NextSeq 500 sequencer (Illumina, San Diego, CA, USA) using a NextSeq 500 mid-output kit V2.5 (300-cycle flow cell) as previously described [6]. 

Briefly, reads were aligned to the GRCh37/hg19 reference genome by a Burrows-Wheeler Aligner (BWA) (v.0.7.17). BAM files were sorted by SAMtools (v.1.7) and purged from duplicates using Mark Duplicates from the Picard suite (v.2.9.0). The GATK’s Haplotype Caller tool (v.4.2) was used to identify variants [7], and these were annotated based on frequency, impact on protein, conservation, and expression using distinct tools, as appropriate (ANNOVAR, dbSNP, 1000 Genomes, EVS, GnomAD, ESP, KAVIAR, and ClinVar) [8,9,10,11,12], and retrieving precomputed pathogenicity predictions with dbNSFP v 3.0 (Poly-Phen-2, SIFT, MutationAssessor, FATHMM, LRT, Revel-score, and CADD) [13] and evolutionary conservation measures. In silico analysis was conducted by running independent algorithms for splice signal detection, including VarSEAK Online Tool (https://varseak.bio, accessed on 7 March 2022), “Ada” and “RF” scores, and SpliceAid (http://www.introni.it/splicing.html, accessed on 7 March 2022). This last tool was applied in order to assess target RNA sequences that are bound by splicing proteins.

Candidate variants were confirmed by Sanger sequencing in both the proband’s and the parents’ DNA. Polymerase chain reaction products were sequenced by employing the BigDye Terminator v1.1 Kit as suggested by the manufacturer (Applied Biosystems, Foster City, CA, USA) and ABI Prism 3100 Genetic Analyzer (Thermo Fisher Scientific, Waltham, MA, USA).

The clinical significance of the identified putative variants was assessed according to the American College of Medical Genetics and Genomics (ACMG) guidelines [14]. 

Variants were prioritized following standardized methods previously described in detail [6].

### 2.3. Functional Assessment of the c.186G>A Variant

RNA was extracted from a buccal swab of the proband and his mother by a MicroRNAeasy kit (Qiagen, Solna, Sweden). Next, it was reversely transcribed using RevertAid First Strand cDNA Synthesis Kit (Thermo Fisher Scientific, Waltham, MA, USA). PCR was performed on the complementary DNA (cDNA) with primers designed on exon 3 and exon 5 (*KMT2E* forward primer: gttgatacagcagagacgtc; and *KMT2E* reverse primer: acttccatcctcagatgtgc). The resulting products were run on 1.5% agarose gel.

## 3. Results

### 3.1. Clinical Description

**Figure 1:**
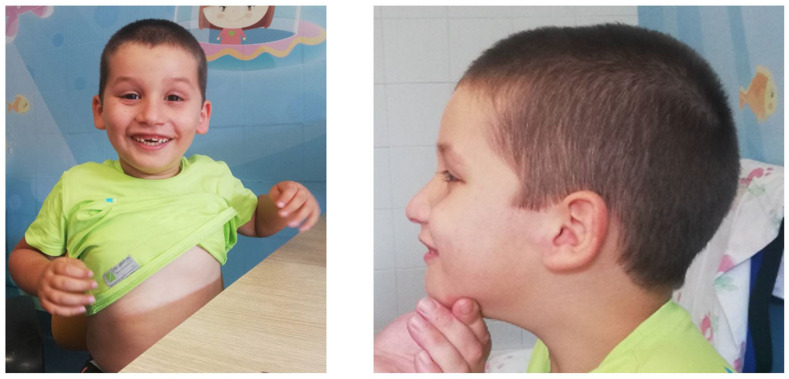
Patient’s clinical features.

The proband is a 6-year-old boy, born at term after an uneventful pregnancy, via vaginal delivery. His height is 122 cm (>75° pc), weight 27.5 kg (50–75° pc for statural age), and CC 55.5 cm (+2 DS for statural age) with dolichocephaly. He presented with horizontal palpebral fissures with bilateral inverted epichantal folds, a wide nasal root, fleshy ear lobes, mild hyperlaxity of the small joints, bilateral clinodactyly of the IV and V digits of the feet, inverted nipples, and a small thoracic hypochromic patch (Figure 1).

The perinatal period was unremarkable. He started walking at 18 months, and he still has difficulties with motor coordination. He did not achieve sphincter control. He spoke his first words at the age of 3, and he is still undergoing speech therapy. He has relational and emotional difficulties; these, together with language impairment, are the main clinical issues. He does not present sleeping or feeding problems. His EEG test was normal, and his brain MRI showed non-specific alterations of the peritrigonal white matter and the posterior part of the lateral ventricles. 

His parents are non-consanguineous and in good health; the proband has an older brother (20 years old), also in good health. The mother had four other pregnancies that resulted in miscarriages for unknown reasons. 

### 3.2. Genetic and Functional Analysis

Preliminary analyses were performed on DNA samples obtained from a blood withdrawal. In particular, an Array-CGH analysis and direct sequencing of the *FMR1* gene were performed, and both were unremarkable.

A TRS analysis of 338 genes causative of syndromic and non-syndromic forms of NDDs allowed us to identify a heterozygous synonymous variant, c.186G>A, p.(Ala62=) in the last nucleotide of exon 4 of the *KMT2E* gene (NM_182931.2). The variant was detected with a depth coverage greater than 150× and with good quality scores (Phread quality > 3000 and genotype quality = 99). Our finding is absent from major databases such as dbSNP, ExAC, 1000 Genomes, and gnomAD. Bioinformatics details are reported in Table 1. 

**Figure 2:**
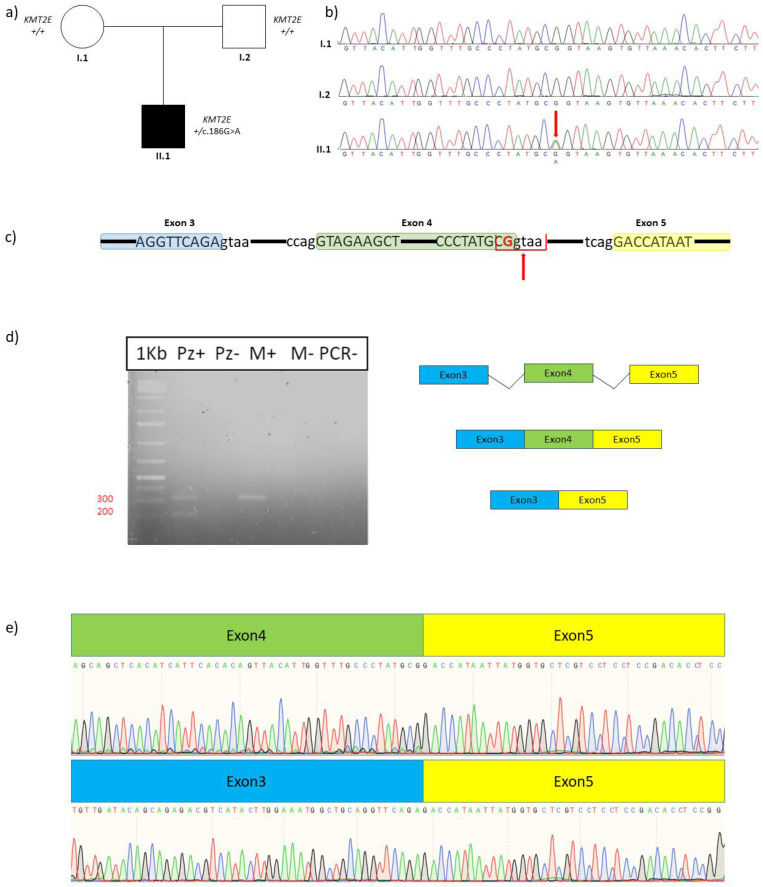
(**a**) Pedigree of the family showing the de novo onset of the variant. Filled and unfilled circles/squares represent affected and unaffected individuals, respectively; (**b**) Electropherograms on DNA of the patient (II.1) and his parents (I.1, I.2). The red arrow indicates the nucleotide substitution; (**c**) Schematic representation of refseq *KMT2E* (NM_182931.2). Blue bar indicates exon 3, green bar indicates exon 4, and yellow bar indicates exon 5. The red arrow indicates the recognition site for ZRANB2 splicing regulatory protein. The final nucleotide of exon 4, where the variant is located, is highlighted by red bold font; (**d**) Gel electrophoresis of the RT–PCR products. Lane 1: marker; Lane 2: Pz+ indicates retrotranscribed patient’s RNA; Lane 3: Pz− indicates not retrotranscribed patient’s RNA (retrotranscription negative control); Lane 4: M+ indicates retrotranscribed patient’s mother RNA; Lane 5: M− indicates not retrotranscribed patient’s mother RNA (retrotranscription negative control); Lane 6: PCR− indicates PCR negative control. The amplification of the mother’s cDNA (M+), as expected, gave a 315 single wt band, while in the proband (PZ+) it showed a wt band and a second band of 215 bp, consistent with the skipping of exon 4; (**e**) Direct sequencing of the isolated bands confirmed the nature of the two alleles, one corresponding to a wt splicing of *KMT2E* exon 3, 4, and 5 (upper sequence), and one allele bearing exon 3 fused with exon 5 (lower sequence).

Subsequently, the variant was confirmed by Sanger sequencing under standard conditions (*KMT2E*, exon 4, forward primer: TGCTAGTTTTCTCAGTGCCG; *KMT2E*, exon 4, reverse primer: GCCGTTTCAAGCACAGTATCA). Segregation analysis was performed in the family showing that it is a de novo event. Functional assessment of the c.186G>A variant was conducted on the cDNA of the proband and his unaffected mother. The amplification of the patient’s cDNA and of his mother gave two different patterns. The amplification of the mother’s cDNA, as expected, gave a clear amplification of a 315 single wild-type band, while the same amplification resulted in two bands in the proband. The second allele identified in the patient was 215 bp, consistent with the skipping of exon 4. Direct sequencing of the isolated bands confirmed the presence of two alleles, one corresponding to a wild-type splicing of exon 3, 4, and 5, and one allele bearing exon 3 fused with exon 5 (Figure 2).

## 4. Discussion

In the present report, we present the clinical description and the molecular characterization of a patient with ODLURO syndrome, recently identified as an emerging form of NDD. Following the initial paper in 2019 by O’Donnell-Luria and collaborators [1], other patients with the condition have been reported, and the evidence shows that individuals with missense mutations usually have a more severe phenotype than those with non-sense mutations or gene deletions, which presents more frequently with severe intellectual disability (ID), drug-resistant epilepsy, and microcephaly instead of macrocephaly [15].

In a recent paper, 18 additional patients with non-sense mutations, splice mutations, or gross deletions were reported. Interestingly, one of the patients, a 13 year-old male proband presenting with speech delay, intellectual disability, constipation, brachymetacarpia, brachymetatarsia, anxiety disorder, selective mutism, and obesity, carried an intronic c.183_186+2del p.(?) de novo variant, predicted to alter splicing at the donor site of exon 4 [3], but the prediction was not confirmed by further studies.

Our patient presents global developmental delay, speech impairment, relational issues, and non-specific brain MRI alterations; he has not experienced epilepsy so far. He carried a heterozygous synonymous variant, c.186G>A, p.(Ala62=), in exon 4 of the *KMT2E* gene (NM_182931.2).

This variant has already been reported in a paper by Yang Li et al. in 2021 [5] in a 5-year-old patient with global developmental delay, autism, social interaction issues, speech impairment, convulsive status epilepticus, EEG alterations, and brain MRI alterations (cerebellar atrophy and broadening of the lateral and third ventricles). The authors analyzed the variant using ensemble scores (“Ada” and “RF”), which predicted the variant to be a splicing donor site loss. Considering these results and the clinical findings compatible with ODLURO syndrome, they suggested that the p.Ala62= variant can be considered pathogenic, but no functional characterization was performed.

Since no functional studies had been conducted, we tried to understand the impact of the c.186G>A variant on the coding sequence on the transcript. The amplification of the patient’s cDNA and that of his mother gave two different patterns. The amplification of the mother’s cDNA, as expected, gave a clear amplification of a 315 single wild-type band, while the same amplification resulted in two bands in the proband. The second allele identified in the patient was 215 bp, consistent with the skipping of exon 4. Direct sequencing of the isolated bands confirmed the presence of two alleles, one corresponding to a wild-type splicing of exon 3, 4, and 5, and one allele bearing exon 3 fused with exon 5.

These results confirm that the c.186G>A (p.Ala62=) variant causes the skipping of exon 4 and further confirm its association with ODLURO syndrome.

Although our patient is the first whose functional investigation has confirmed the deleterious consequence of the reported variant, animal models could be useful to corroborate this emerging molecular evidence and to help our understanding of disease mechanisms and pathophysiology.

Furthermore, we investigate the possible molecular mechanism involved in the exon skipping of exon 4. Pre-mRNA splicing is the process of removing introns from primary transcripts. Alternative splicing, the process by which a single primary transcript yields different mature mRNAs, can lead to the production of several protein isoforms. These isoforms can have different functions due to changes in binding properties, enzymatic activity, protein stability, or intracellular localization. Pre-mRNA splicing is performed by the spliceosome, consisting of five small nuclear ribonuclear protein particles (snRNPs) and hundreds of proteins, including the human zinc finger splicing factor. One of them, the human zinc finger ranbp2-type domain-containing protein 2 (ZRANB2), has two zinc finger domains, a glutamic acid-rich region and a C-terminal Ser/Arg-rich (SR) domain, and as such resembles members of the SR-related family of splicing factors [16]. The two N-terminal zinc fingers (ZnFs) of ZRANB2 recognize single-stranded (ss) RNA with high affinity and specificity. This suggests that the regulation of alternative splicing by ZRANB2 occurs via a direct interaction with pre-mRNA at sites that resemble the consensus 5′ splice site [17]. Loughlin et al. demonstrated that the standard binding site on ssRNA of ZRANB2 is the AGGUAA motif [17]. They also measured the affinity of each zinc finger for a series of ssRNA oligonucleotides that each contained a single purine-to-pyrimidine or pyrimidine-to-purine mutation in the AGGUAA motif. ZRANB2 was capable of binding the mutated motifs with similar affinity, except for mutations involving the central GG sequence, which resulted in being the most important for binding.

Based on in silico prediction by the bioinformatics tool “Introni” (SpliceAid) [18], the identified binding motif in our case is CGGUAA. Based on this previously reported evidence, we suggest that the c.186G>A variant disrupts/modifies the essential GG binding site of the ZRANB2 protein. Consequently, this alteration is expected to induce an aberrant splicing process, resulting in the skipping of exon 4.

## 5. Conclusions

Next-generation sequencing is a crucial tool for diagnosing neurodevelopmental disorders. In this study, we describe a male patient with ODLURO syndrome carrying a synonymous variant in the *KMT2E* gene, with its functional evaluation never performed to date. This mutation hinders the process of splicing, leading to the exclusion of exon 4 from mRNA and the production of a truncated protein. We propose a possible molecular mechanism underlying the aberrant splicing, but further studies are necessary to corroborate this hypothesis. Furthermore, studies on patients with novel variants in the *KMT2E* gene are necessary to better characterize the associated clinical features and molecular mechanisms that play a role in the onset of disease. An early and correct diagnosis has the potential to reduce frustration and anxiety within the family, as well as support family planning, even if there is no specific treatment currently available.

## Figures and Tables

**Table 1:** Bioinformatics details of *KMT2E* variant.

Gene	*KMT2E*
Refseq	NM_182931.3
Nucleotide change	c.186G>A
Amino acid change	p.(Ala62=)
Genotype	Heterozygous
Inheritance	De novo
GnomAD frequency	N.A.
TOPmed frequency	N.A.
1000 Genomes frequency	N.A.
dbscSNV Ada	1 Deleterious
dbscSNV RF	0.88 Deleterious
Varseak	Class 4. Likely loss of function for authentic Splice Site. Exon skipping
Introni (SpliceAid)	No binding sites CGGUAA for ZRANB2

N.A., not available.

## Data Availability

The data presented in this study are reported in the Leiden Open Variation Database (LOVD) (https://databases.lovd.nl/shared/variants/0000921776#00025825) (accessed on 21 November 2023).
